# Activity of Nonhallucinogenic Ibogalogs on Chemotherapy-Induced Peripheral Neuropathic Pain in Mice

**DOI:** 10.1021/acschemneuro.5c01002

**Published:** 2026-05-01

**Authors:** Jason Younkin, Eda Koseli, Belle Buzzi, Edward Choi, Torin Honaker, Randall L. Davis, Dina Manetti, Maria Novella Romanelli, Aysel Cetinkaya-Fisgin, Hugo R. Arias, Ahmet Hoke, Javier Gonzalez-Maeso, M. Imad Damaj

**Affiliations:** † Department of Pharmacology and Toxicology, 6889 Virginia Commonwealth University, Richmond, Virginia 23298, United States; ‡ Department of Pharmacology and Physiology, Oklahoma State University − Center of Health Sciences, Tulsa and Tahlequah, Oklahoma 74107, United States; § Department of Neurosciences, Psychology, Drug Research and Child Health Section of Pharmaceutical and Nutraceutical Sciences, 60222 University of Florence, Sesto Fiorentino 50019, Italy; ∥ Department of Neurology, The Johns Hopkins University School of Medicine, Baltimore, Maryland 21205, United States; ⊥ Department of Biology, Virginia State University, Petersburg, Virginia 23806, United States

**Keywords:** ibogalogs, chronic pain, neuropathy, 5-HT_2A_R, mice, chemotherapy

## Abstract

In this study, we examined the effects of ibogaminalog (DM506), a nonhallucinogenic analog of ibogamine, in a mouse model of chemotherapy-induced peripheral neuropathy (CIPN) induced by paclitaxel. We also assessed the effects of tabernanthalog (TBG) and investigated the potential role of serotonin receptor subtype 2A (5-HT_2A_R) for both compounds. DM506 and TBG demonstrated antinociceptive activity in the CIPN model. DM506 produced a more prolonged effect, lasting between 10 and 14 days, with nociception reduction in mechanical (von Frey) and cold (acetone) hypersensitivity assays. These effects were observed in a dose- and time-dependent manner. Unlike some previous studies that reported effects lasting only hours, the effects we observed persisted for several days. Furthermore, neither compound produced long-lasting effects on locomotor activity, even at relatively high doses. The antinociceptive effects of both compounds in the CIPN mouse model were blocked by the 5-HT_2A_R selective antagonist volinanserin. *In vitro* studies revealed that DM506 does not block cytokine/chemokine expression in microglial cells but partially protected dorsal root ganglion (DRG) neurons treated with paclitaxel in the 100–300 nM concentration range in a volinanserin-sensitive manner. We conclude that DM506 and TBG possess antinociceptive properties in mice via pathways involving 5-HT_2A_R activation. Although it is unlikely that this effect involves anti-inflammatory activity in microglia, partial neuroprotective activity in DRG neurons can be considered.

## Graphical abstract

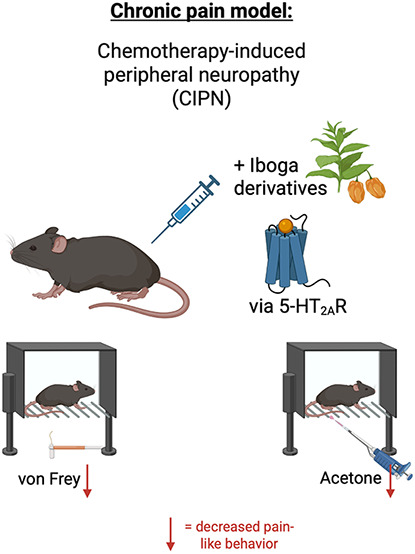

## 1 Introduction

Chronic pain remains a critical public health challenge worldwide, affecting approximately one in five adults globally. In the United States, recent data from the Centers for Disease Control and Prevention (CDC) show that the prevalence of chronic pain was at 24.3% in 2023.^1^ The management of chronic pain, particularly for neuropathic pain, is very challenging. Current first-line treatments, such as gabapentinoids and tricyclic antidepressants, often provide only a modest efficacy and are frequently limited by dose-dependent side effects. Furthermore, many potent analgesics like opioids carry significant risks, including high abuse liability and the potential for fatal overdose.^2^ There is an urgent need to develop more efficacious and safer treatments for chronic neuropathic pain.

Novel nonhallucinogenic analogs of iboga alkaloids, including tabernanthalog (TBG) and ibogaminalog (DM506), were recently reported to show efficacy in animal models of addiction and depression- and anxiety-like behaviors via activation of the serotonin receptor subtype 2A (5HT_2A_R).^3–6^ In addition, DM506 and TBG also possess antinociceptive properties in mouse models of neuropathic (chronic constriction injury and oxaliplatin models) and visceral pain (dextran sulfate sodium model) with no obvious behavioral toxicity.^7,8^ DM506’s effects in these pain models were blocked by ketanserin, a 5-HT_2A/2C_R antagonist,^9^ suggesting an important contribution of these receptors to DM506’s antinociceptive efficacy in mice. DM506 and TBG have a complex pharmacological profile. In vitro functional studies showed that both compounds are potent agonists at the 5-HT2AR, 5-HT2CR, and 5-HT6R subtypes, whereas DM506 behaves as a 5-HT2BR agonist and TBG functionally acts as a competitive antagonist.^3,5–8,24^ In addition, both ibogalogs are inverse agonists at the 5-HT7R, allosteric inhibitors of α7 and α9α10 nicotinic acetylcholine receptors, and inhibitors of the serotonin transporter (SERT) and the α2A-adrenergic receptor.^3,7,10,11^

In this study, we further explored the effects of DM506 on neuropathic pain by examining its effects in an animal model of chemotherapy-induced peripheral neuropathy (CIPN) in male and female mice. We determined the binding affinity of DM506 at the 5-HT_2A_R using [^3^H]­ketanserin competition binding experiments. To assess the potential hallucinogenic-like effects of DM506, we performed the head-twitch response (HTR) in mice, a proven correlative to hallucinations in humans.^12^ We then compared the efficacy of DM506 and TBG in the CIPN mouse model. We also examined whether the antinociceptive effects of DM506 and TBG are blocked by volinanserin, a potent and more selective 5-HT_2A_R antagonist.^9^ Finally, we assessed whether DM506 has anti-inflammatory activity in microglial cells treated with paclitaxel.

## 2 Materials and Methods

### 2.1 Animals

The experiments were performed on C57BL/6J adult female and male mice 8–12 weeks old purchased from The Jackson Laboratory (Bar Harbor, ME). Animals were housed individually with an enriched environment and maintained on a 12 h light/dark cycle (lights on at 7:00 AM), with a room temperature of 22 °C and *ad libitum* access to food (global 18% protein chow diet; Envigo Teklad, Indianapolis, IN, USA) and water. All experiments were performed during the light cycle. The study was approved by the Institutional Animal Care and Use Committee of Virginia Commonwealth University (Protocol # 10142). All studies were carried out following the National Institutes of Health’s Guide for the Care and Use of Laboratory Animals and complied with ARRIVE (Animal Research: Reporting of In Vivo Experiments) guidelines. All mice were observed daily for general well-being, and their weight was measured daily.

### 2.2 Drugs

[^3^H]­Ketanserin (specific activity of 67 Ci/mmol) was obtained from PerkinElmer Life and Analytical Sciences (Waltham, Massachusetts). The drugs used in this study were purchased from the following sources: Paclitaxel (Athenex, NDC 70860-200-50, Richmond, VA, USA; Biosynth International Inc., San Diego, CA, USA; and Sigma-Aldrich, St. Louis, MO, USA), serotonin hydrochloride, DOI, volinanserin, acetone, ethanol, forskolin, and kolliphor (Sigma-Aldrich, St. Louis, MO, USA). ELISA kits for IL-6 and CCL2 were obtained from Thermo Fisher Scientific (Waltham, MA, USA). DMEM/Ham’s F-12 was purchased from Corning (Glendale, AZ, USA). FBS (fetal bovine serum), l-glutamine, penicillin, and streptomycin were purchased from Gibco BRL (San Francisco, CA, USA). Methysergide was obtained from Tocris Bioscience (Minneapolis, MN, USA). Pierce BCA protein assay kit was purchased from Thermo Fisher Scientific (Waltham, MA, USA). GF/C glass fiber filters and polyethylenimine were obtained from PerkinElmer (Springfield, IL). DM506 (3-methyl-1,2,3,4,5,6-hexahydroazepino­[4,5-*b*]­indole fumarate) was synthesized as previously reported.^11^ TBG (8-methoxy-3-methyl-1,2,3,4,5,6-hexahydroazepino­[4,5-*b*]­indole fumarate) was synthesized based on the procedure described previously.^3^

### 2.3 Radioligand Affinity Binding

As previously described, radioligand affinity binding studies were performed using membrane preparations from HEK293 cells stably expressing ∼260 fmol/mg protein of human 5-HT_2A_Rs.^13,14^ Cell pellets were homogenized with a Teflon-glass pestle (≥50 up-and-down strokes) in 7 mL of binding buffer (50 mM Tris-HCl; pH 7.4). The volume was increased to 10 mL with binding buffer, and the crude homogenate was centrifuged at 1,000×*g* for 5 min at 4 °C. The supernatant was centrifuged at 40,000×*g* for 15 min. The remaining pellet was washed with 10 mL of binding buffer and centrifuged at 40,000×*g* for 15 min. Protein concentration was determined using the Pierce BCA protein assay kit. Competition concentration–response curves were obtained by incubating each compound (1 nM-1 mM; dissolved in physiological saline) with binding buffer containing 5 nM [^3^H]­ketanserin and 32–38 μg of protein. The final volume in each well was 200 μL. Nonspecific binding was determined using 10 μM methysergide. Free ligand was separated from bound ligand by rapid filtration under vacuum through GF/C glass fiber filters that were presoaked in 0.5% polyethylenimine using a MicroBeta Filtermat-96 harvester (PerkinElmer). The filters were then rinsed with ice-cold incubation buffer and dried at 65 °C for 1 h. The radioactivity on the filters was counted by using a MicroBeta2 detector (PerkinElmer). Binding data were analyzed by Excel and then by nonlinear regression with GraphPad Prism (GraphPad Software, La Jolla, CA).

### 2.4 Head-Twitch Response (HTR)

HTR assays using magnetic ear-tagging were performed as previously reported.^15,16^ Testing occurred no more than once per week with at least 7 days between test sessions. On test days, mice were placed individually into the coiled chamber for 30 min to acclimate to the environment and determine baseline HTR. Subsequently, DOI (1 mg/kg, i.p.), DM506 fumarate (40 mg/kg, i.p.) (both dissolved in physiological saline), or vehicle was administered immediately prior to HTR recording for a 30 min session. These doses were based on previous studies with these two compounds.^6,17^

### 2.5 Chemotherapy-Induced Peripheral Neuropathy (CIPN) Pain Model

Paclitaxel was dissolved in a 1:1:18 mixture of 200 proof ethanol, kolliphor, and distilled water (i.e., vehicle). Paclitaxel was administered at a dose of 8 mg/kg intraperitoneally every other day; four administrations completed one injection cycle. Control mice received vehicle at a volume of 10 mL/kg, i.p., and followed the same injection cycle. Animals began behavioral testing 24 h after the final injection. DOI, volinanserin, DM506 fumarate, and TBG fumarate were dissolved in physiological saline, and solutions were administered subcutaneously (s.c.) (volinanserin) or i.p. (the rest of the drugs) at 10 mL/kg.

#### 2.5.1 CIPN Experiments

On day 28 after paclitaxel, different cohorts of mice were injected (i.p.) with a single dose of DM506 fumarate [10 and 40 mg/kg, corresponding to 7.8 and 31 mg/kg of DM506 (base)] or TBG fumarate [20 and 40 mg/kg, corresponding to 13 and 27 mg/kg of TBG (base)] (dissolved in physiological saline). Mechanical (von Frey) and cold (acetone) sensitivity tests were evaluated at different time points for each drug for up to 14 days.

#### 2.5.2 5-HT_2A_R Antagonist Experiments

In a separate cohort of mice, we used volinanserin to block the 5-HT_2A_R. On day 28, after paclitaxel administration, different cohorts of mice were injected with volinanserin (0.05 mg/kg, s.c., dissolved in physiological saline) 15 min prior to a single dose of DM506 fumarate (40 mg/kg, i.p.) or TBG fumarate (40 mg/kg, i.p.). Volume injections were at 10 mL/kg. Mechanical (von Frey) and cold (acetone) sensitivity tests were evaluated at 1, 3, 6, and 24 h (TBG) or at 6 h, and days D1, D7, D10, and D14 (DM506) after drug injection.

### 2.6 Mechanical Sensitivity Test

Mechanical withdrawal thresholds were determined using von Frey filaments as previously described.^18^ Mice were acclimated to a Plexiglas cage on mesh metal flooring for 30 min prior to testing. Withdrawal thresholds were measured by applying a series of calibrated von Frey filaments (Stoelting, Wood Dale, IL; logarithmically incremental force from 2.83 to 5.88 expressed in dsLog 10 of [10 pound force in milligrams]) to the hind paw. Using a modified up–down method, in the absence of a paw withdrawal response (paw withdrawn, licking, or shaking) to the initially selected filament, a thicker filament corresponding to a stronger stimulus was presented. Once paw withdrawal occurred, the next weaker stimulus was chosen. Each filament was presented vertically against the paw, with sufficient force to cause slight bending, and held for 2–3 s. Stimulation of the same intensity was applied 3 times at intervals of a few seconds.

### 2.7 Cold Sensitivity Test

Cold hypersensitivity was assessed by using an acetone test. Mice were placed individually into a cage with mesh metal flooring (Bioseb-PVF, Bioseb, Chaville, France) for 30 min prior to testing. Twenty μL of acetone was applied onto the plantar surface of each hind paw via pipette. Time spent licking, shaking, or shaking the hind paw was recorded for 60 s.

### 2.8 Locomotor Activity

Mice were placed into individual Omnitech photocell activity cages (28 × 16.5 cm; Columbia, OH, USA) 6 and 24 h after administration of either vehicle or DM506 fumarate (40 mg/kg, i.p.). Interruptions of the photocell beams (two banks of eight cells each) were then recorded over 60 min. Data are expressed as the number of photocell interruptions.

### 2.9 Assessment of Neuroprotective Effect of DM506 on Dorsal Root Ganglion (DRG) Neurons

Assessment of *in vitro* neuroprotection was performed using primary embryonic rat dorsal root ganglia (DRG) neuron–Schwann cell cocultures as previously described.^19^ Briefly, DRGs were aseptically dissected and isolated from Sprague–Dawley rat embryos on embryonic day 15 (E15). The cells were enzymatically dissociated using collagenase and trypsin and then suspended in Neurobasal medium (Gibco, 21103-049) supplemented with 1% fetal bovine serum (FBS; Hyclone, 3H30071.3), GlutaMAX (Gibco, 35050061), 10 ng/mL glial cell line-derived neurotrophic factor (GDNF; Peprotech, 450-51), 2% B27 supplement (Gibco, 17504-044), and 0.2% glucose. Sterile 96-well tissue culture plates were precoated with 100 μg/mL poly-d-lysine hydrobromide (Sigma-Aldrich, P7280), followed by 10 μg/mL laminin (Invitrogen, 23017-015). DRG cells were seeded at a density of 3,500 cells per well and incubated in a humidified incubator at 37 °C with 5% CO_2_. Neurite outgrowth and cellular differentiation were assessed after 24 h using an inverted light microscope. DM506 was added at various concentrations, either alone or in combination with paclitaxel, and cells were incubated for an additional 24 h. NAD^+^ levels were measured using the NAD/NADH-Glo Assay (Promega) according to the manufacturer’s protocol. The assay was repeated with volinanserin at a 1 μM concentration.

### 2.10 Assessment of DM506’s Effects on Paclitaxel-Treated C20 Microglial Cells

Immortalized human C20 microglial cells^20^ were cultured in DMEM/Ham’s F-12 (1:1) medium supplemented with 2.5 mmol/L l-glutamine, 10% FBS, and 1% penicillin/streptomycin. Growth medium was replaced at 48 h intervals. Cells were seeded in 24-well plates (1.15 × 10^4^ cells/well) and cultured until 90% confluent as previously described.^21^ Cells were then cultured overnight in a serum-free medium. Preliminary experiments showed that 10 μM paclitaxel stimulates microglia and that DM506 does not decrease cell viability at concentrations up to 100 nM. Subsequently, cells were either unstimulated (i.e., control cells) or stimulated with 10 μM paclitaxel and then immediately treated with 10 nM DM506 or vehicle (0.1% DMSO) for 24 h. Considering that DM506 is a potent agonist of the 5-HT_2A_R,^5^ we tested whether ketanserin, a potent 5-HT_2_R antagonist,^9^ may inhibit DM506-induced effects. In this regard, cells were preincubated with 1 μM ketanserin for 30 min before cotreatment with paclitaxel and DM506 as described above. Following the 24 h treatment period, the culture medium was collected and stored at −80 °C until further analysis. Levels of cytokines IL-6 and CCL2 secreted into the medium were quantitated by ELISA, following the source’s protocol. These values were subsequently normalized to total cell protein, determined by the BCA assay as previously described.^21^

### 2.11 Statistical Analysis

Data were analyzed using GraphPad Prism software (version 9.3 or 10.1.0, GraphPad Software, Inc., La Jolla, CA). Normality and equal variance have been verified by Shapiro-Wilk and Brown-Forsythe tests. Data were then compared using either one- or two-way analysis of variance (ANOVAs) with repeated measures, followed by different post hoc comparison tests. Data are expressed as the mean ± SEM of mice per group for all tests. The number of experiments is indicated in each figure legend. The *p*-values less than 0.05 were considered significant. Because sex was not a significant variable, data from male and female mice were pooled for subsequent behavioral analyses.

## 3 Results

### 3.1 DM506 Binds at the 5-HT_2A_R but Does Not Induce HTR

To ensure that DM506 binds to the 5-HT_2A_R, we examined the affinity of DM506 for the 5-HT_2A_R by displacement of [^3^H]­ketanserin binding in membrane preparations from HEK293 cells stably expressing the human 5-HT_2A_R (Figure 1A) [*pK*_i_ = −6.889 ± 0.116 (129 ± 34 nM)]. We then tested the ability of DOI (1 mg/kg) and DM506 (40 mg/kg) to produce HTR, a behavioral signature of psychedelic drugs upon activation of 5-HT_2A_Rs,^12^ in male and female C57BL/6J mice. As expected, DOI produced a significant increase in the number of HTR compared to the saline control (Figure 2A,B). A two-way ANOVA analysis of time [F_(1.574, 11.02)_ = 5.255; *p* = 0.0037], treatment [F_(2, 7)_ = 13.36; *p* = 0.0041], and time × treatment [F_(6, 21)_ = 10.59; *p* < 0.0001] were significant, and a post hoc Dunnett test revealed that DOI at 15 and 30 min after injection was significantly higher than the vehicle group. One-way ANOVA analysis of the total number of HTRs induced by DOI was significantly higher [F_(2, 15)_ = 44.01; *p* < 0.0001] compared to the vehicle group. However, no significant increase in the number of HTRs was seen after DM506 injection.

**Figure 1:**
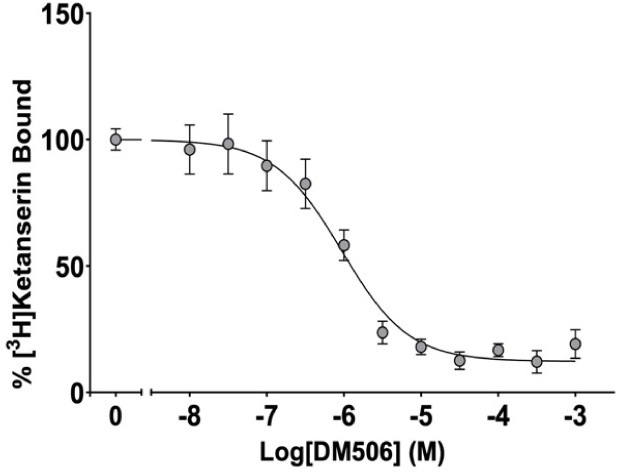
Radioligand binding for DM506 at the 5-HT_2A_R. [^3^H]­Ketanserin binding displacement (*n* = 3–4 independent experiments performed in duplicate) on membrane preparations from HEK293 cells stably expressing human 5HT_2A_Rs. [*pK*_i_ = −6.889 ± 0.116 (129 ± 34 nM)].

**Figure 2:**
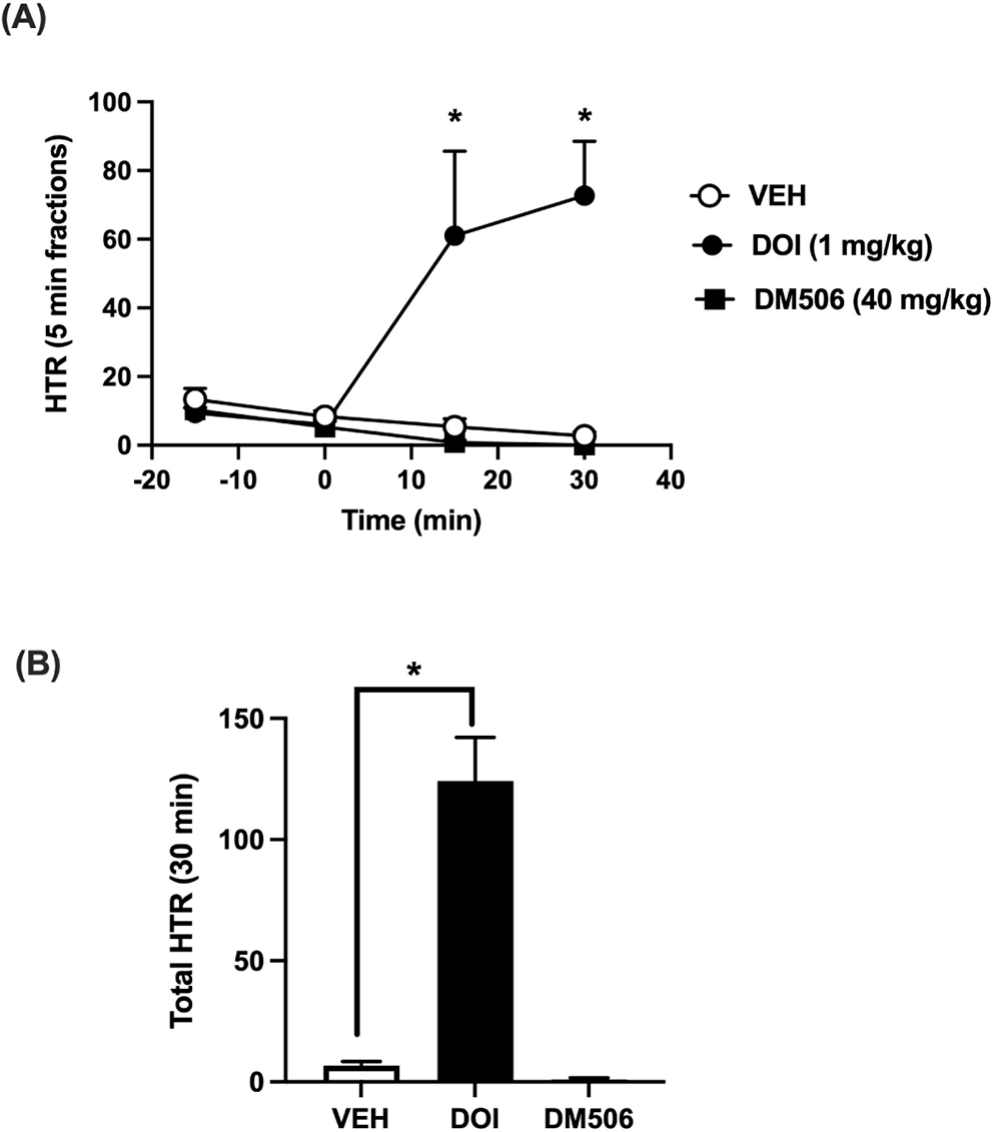
Effect of DM506 and DOI on HTR in C57BL/6J mice. (A) Time-course showing HTR counts in 15 min blocks corresponding to doses of DM506 fumarate (40 mg/kg), DOI (1 mg/kg), or vehicle (VEH). (B) Sum of HTR during the first 30 min after DM506, DOI, or vehicle administration. Values are expressed as mean ± SEM, *n* = 8/group (F+M), * *p* < 0.05 versus VEH.

### 3.2 DM506 Reduces Paclitaxel-Induced Mechanical and Cold Hypersensitivity after a Single I.P. Injection

We first tested whether DM506 would reverse paclitaxel-induced neuropathic pain in male and female mice. Mice were injected acutely with DM506 at different doses (10 and 40 mg/kg; i.p.) on day 28 after the first paclitaxel injection, and mechanical (von Frey) and cold hypersensitivity (acetone) were assessed at different time points (at 6 h and on days D1, D7, D10, and D14) after DM506 injection.

A two-way ANOVA analysis of the mechanical hypersensitivity data showed a significant treatment [F_(4, 35)_ = 85.86; *p* < 0.0001], time [F_(5.229, 183.0)_ = 44.1; *p* < 0.0001], and treatment × time [F_(24, 210)_ = 10.33; *p* < 0.0001] effect (Figure 3). Paclitaxel treatment induced mechanical hypersensitivity at all time points tested (*p* < 0.001; Figure 3A). In addition, DM506 injection was able to reverse paclitaxel-induced mechanical hypersensitivity in a time- and dose-dependent manner [Paclitaxel/Veh vs Paclitaxel/DM506 10 mg/kg: 6 h, D1, D7, and D10 (*p* < 0.05); 40 mg/kg: 6 h, D1, D7, and D10 (*p* < 0.01); Figure 3A].

**Figure 3:**
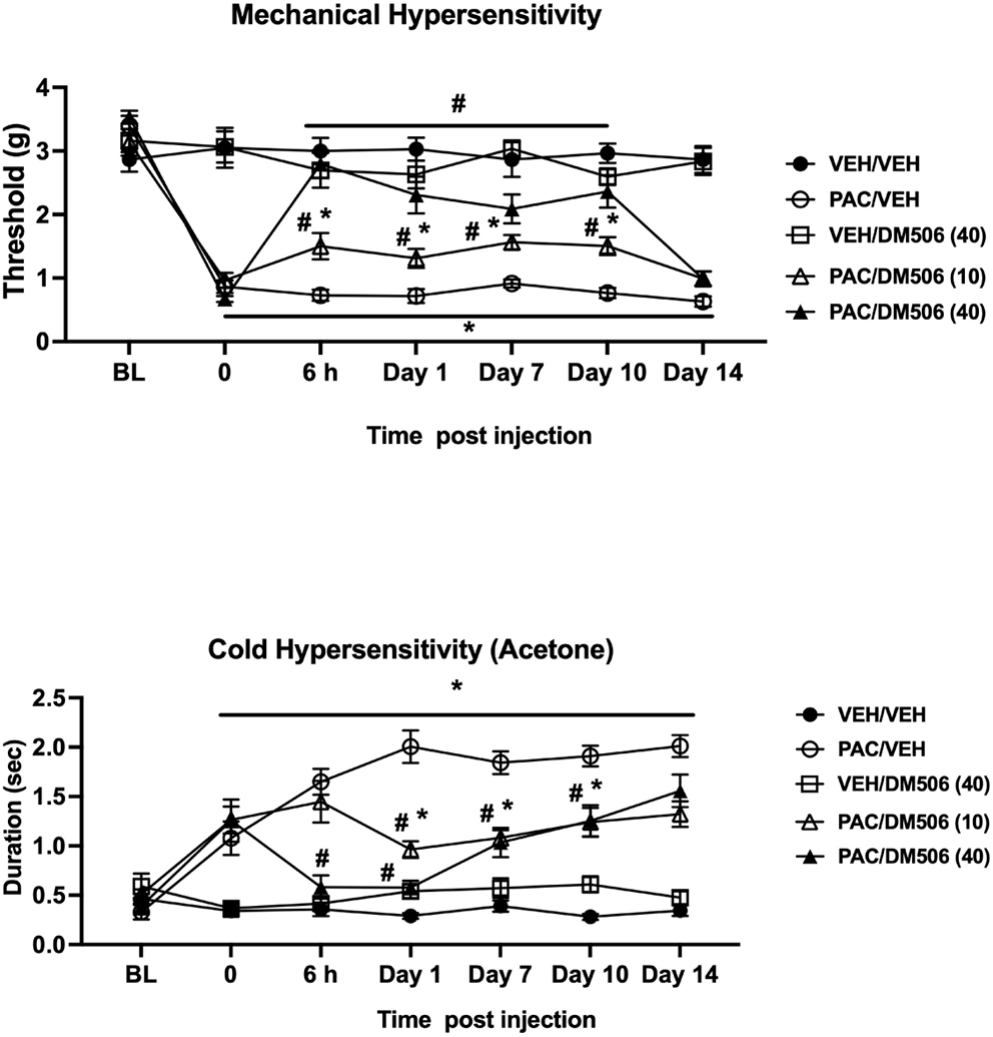
DM506 dose-dependently reversed chemotherapy-induced mechanical and cold hypersensitivity in mice. Acute dosing of DM506 fumarate (10 and 40 mg/kg, i.p.) reversed chemotherapy-induced neuropathic pain in mechanical and cold hypersensitivity assays for up to 14 days after drug administration. Values are expressed as mean ± SEM, *n* = 8/group (F+M). Results were analyzed using two-way ANOVA followed by a post hoc Tukey test (# vs PAC/VEH, * vs VEH/VEH; *p* < 0.05). PAC = paclitaxel; VEH = vehicle.

Similarly, assessment of cold hypersensitivity demonstrated, through two-way ANOVA, a significant treatment [F_(4, 35)_ = 71.00; *p* < 0.0001], time [F_(4.889, 171.1)_ = 21.87; *p* < 0.0001], and treatment × time [F_(24, 210)_ = 10.25; *p* < 0.0001] effect (Figure 3B). Paclitaxel treatment induced cold hypersensitivity at all time points tested (*p* < 0.05; Figure 3B). However, DM506 injection was able to reverse paclitaxel-induced cold hypersensitivity in a time- and dose-dependent manner [Paclitaxel/Veh vs Paclitaxel/DM506 10 mg/kg: 6 h, D1, D7, D10, and D14 (*p* < 0.05); 40 mg/kg: 6 h, D1, D7, and D10 (*p* < 0.05); Figure 3B].

### 3.3 TBG Reduces Paclitaxel-Induced Mechanical and Cold Hypersensitivity after a Single I.P. Injection

We then assessed the effects of TBG after a single acute injection in a different cohort of the same mouse pain model. Mice were injected acutely with TBG at doses of 20 and 40 mg/kg, i.p., on day 28 after the first paclitaxel injection, and mechanical (von Frey) and cold hypersensitivity (acetone) were assessed at different time points (1, 3, 6, and 24 h) after TBG injection. A two-way ANOVA analysis of the mechanical hypersensitivity data showed a significant treatment [F_(5, 42)_ = 60.75; *p* < 0.0001], time [F_(4, 175)_ = 18.45; *p* < 0.0001], and treatment × time [F_(25, 210)_ = 5.297; *p* < 0.0001] effect (Figure 4A). While the TBG administration was able to reverse paclitaxel-induced mechanical hypersensitivity in a dose-related manner, its effects lasted for only 24 h after injection [Paclitaxel/Veh vs Paclitaxel/TBG 20 mg/kg: 1, 3, 6, and 24 h (*p* < 0.05); 40 mg/kg: 1, 3, 6, and 24 h (*p* < 0.05); Figure 4A].

**Figure 4:**
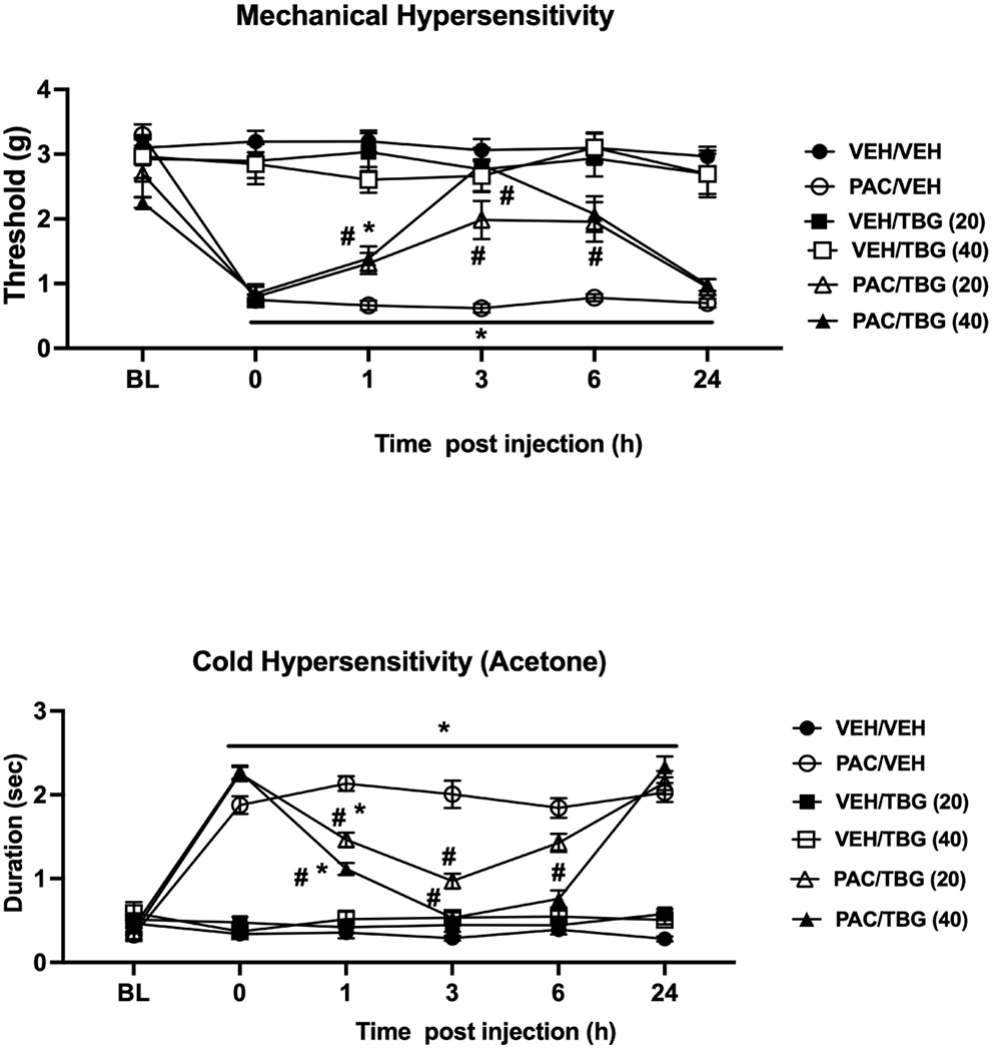
TBG dose-dependently reversed chemotherapy-induced mechanical and cold hypersensitivity in mice. Acute dosing of TBG fumarate (20 and 40 mg/kg, i.p.) reversed chemotherapy-induced neuropathic pain in mechanical and cold hypersensitivity assays after drug administration. Values are expressed as mean ± SEM, *n* = 8/group (F+M). Results were analyzed using two-way ANOVA followed by a post hoc Tukey test (# vs PAC/VEH, * vs VEH/VEH; *p* < 0.05). PAC = paclitaxel; VEH = vehicle.

A similar profile was observed with cold hypersensitivity, demonstrated after TBG administration. The two-way ANOVA analysis showed a significant treatment [F_(5, 42)_ = 186.1; *p* < 0.0001], time [F_(4, 175)_ = 94.23; *p* < 0.0001], and treatment × time [F_(25, 210)_ = 33.93; *p* < 0.0001] effect (Figure 4B). TBG injection was able to reverse paclitaxel-induced cold hypersensitivity in a time- and dose-dependent manner [Paclitaxel/Veh vs Paclitaxel/TBG 20 mg/kg: 1 and 3 h (*p* < 0.05), but not at 6 and 24 h; 40 mg/kg: 1, 3, and 6 h (*p* < 0.05), but not at 24 h; Figure 4B].

### 3.4 The Antinociceptive Effects of DM506 and TBG Are Mediated via the 5-HT_2A_R

Since both DM506 and TBG are agonists at the 5-HT_2A_R,^6^ we tested whether volinanserin (a selective 5-HT_2A_R antagonist)^9^ would block their antinociceptive effect. We pretreated a separate cohort of mice with volinanserin before DM506 injection in the CIPN mouse model. On day 28, after the first paclitaxel injection, different groups of mice were injected with either volinanserin (0.05 mg/kg, s.c.) or vehicle 15 min before a single dose of DM506 (40 mg/kg, i.p.) or TBG (40 mg/kg, i.p.). Mechanical (von Frey) and cold (acetone) hypersensitivity tests were evaluated at 1, 3, 6, and 24 h (TBG) or at 6 h and days D1, D7, D10, and D14 (DM506) after drug injection.

A two-way ANOVA analysis of the mechanical hypersensitivity data showed a significant treatment [F_(5, 42)_ = 9.305; *p* < 0.0001], time [F_(1, 42)_ = 70.03; *p* < 0.0001], and treatment × time [F_(5, 42)_ = 12.22; *p* < 0.0001] effect (Figure 5A). DM506 (40 mg/kg) was able to fully reverse paclitaxel-induced mechanical hypersensitivity 24 h after injection (*p* < 0.0001; Figure 5A). In addition, the effect of DM506 was completely blocked by volinanserin pretreatment (*p* < 0.0001; Figure 5A). The effect of DM506 on the cold hypersensitivity measures was also blocked by volinanserin. A two-way ANOVA analysis of cold hypersensitivity demonstrated a significant treatment [F_(5, 42)_ = 38.94; *p* < 0.0001], time [F_(1, 42)_ = 114.0; *p* < 0.0001], and treatment × time [F_(5, 42)_ = 42.26; *p* < 0.0001] effect (Figure 5B). DM506 (40 mg/kg) was able to fully reverse paclitaxel-induced cold hypersensitivity 24 h after injection (*p* < 0.0001; Figure 5B). Furthermore, the effect of DM506 was completely blocked by volinanserin pretreatment (*p* < 0.0001; Figure 5B).

**Figure 5:**
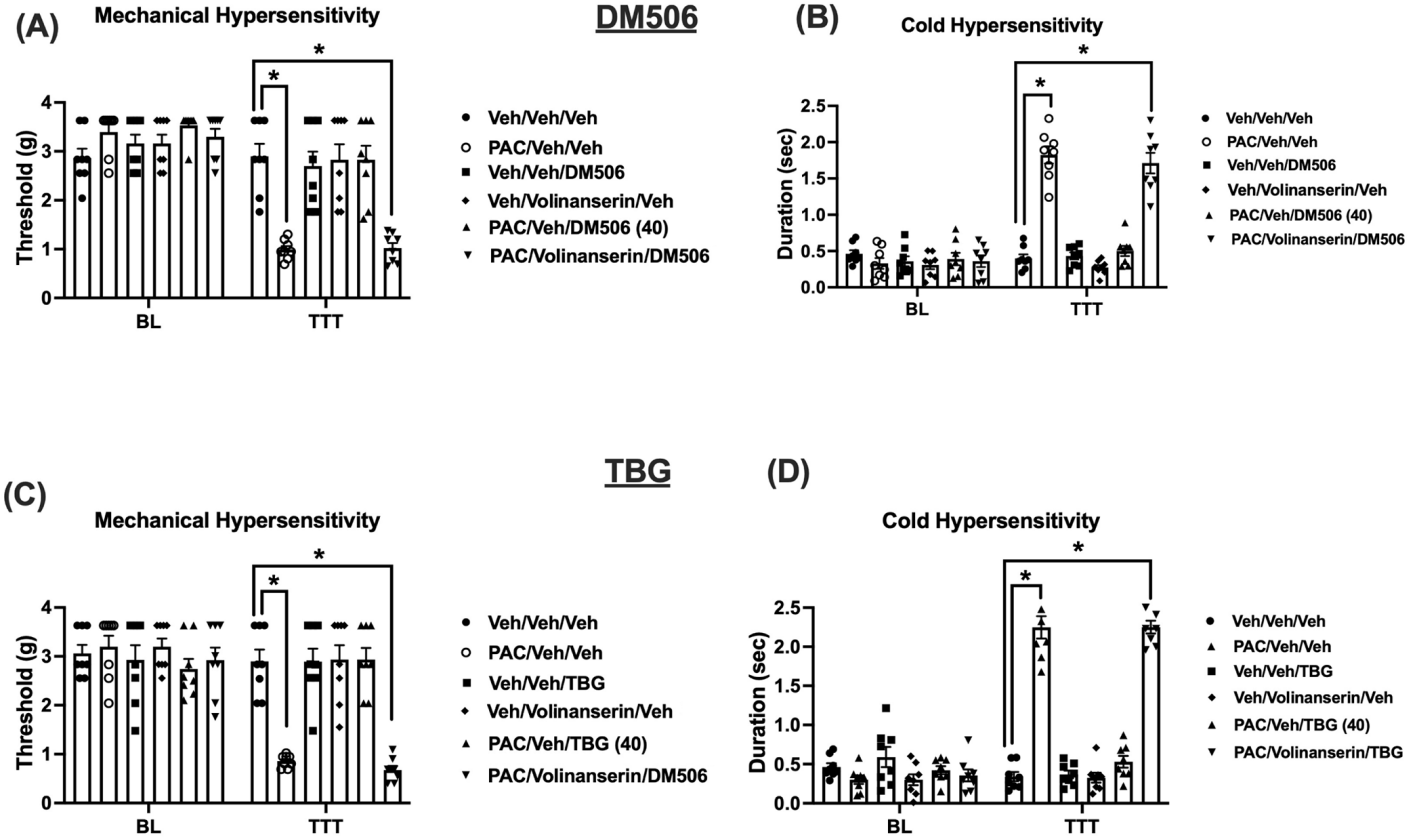
The behavioral effects of DM506 and TBG are mediated through 5HT_2A_R activation. Volinanserin blocked the reversal effect of DM506 fumarate (A, B) and TBG fumarate (C, D) (40 mg/kg each) on chemotherapy-induced mechanical and cold hypersensitivity in mice. Values are expressed as mean ± SEM, *n* = 8/group (F+M). Results were analyzed using two-way ANOVA followed by a post hoc Tukey test (* indicates *p* < 0.05). PAC = paclitaxel; Veh = vehicle; BL = Baseline; TTT = Treatment.

Similarly, the effects of TBG were completely blocked by the 5HT_2A_R antagonist volinanserin. A two-way ANOVA analysis of the mechanical hypersensitivity data showed a significant treatment [F_(5, 42)_ = 12.23; *p* < 0.0001], time [F_(1, 42)_ = 40.03; *p* < 0.0001], and treatment × time [F_(5, 42)_ = 13.87; *p* < 0.0001] effect (Figure 5C). TBG (40 mg/kg) was able to fully reverse paclitaxel-induced mechanical hypersensitivity 3 h after injection (*p* < 0.0001; Figure 5C) and its effect was completely blocked by volinanserin pretreatment (*p* < 0.0001; Figure 5C). The effects of TBG in the cold hypersensitivity measures were also blocked by volinanserin. A two-way ANOVA analysis of cold sensitivity demonstrated a significant treatment [F_(5, 42)_ = 63.69; *p* < 0.0001], time [F_(1, 42)_ = 169.8; *p* < 0.0001], and treatment × time [F_(5, 42)_ = 81.26; *p* < 0.0001] effect (Figure 5D). TBG (40 mg/kg) was able to fully reverse paclitaxel-induced cold hypersensitivity 3 h after injection (*p* < 0.0001; Figure 5D). In addition, the effect of TBG was completely blocked by volinanserin pretreatment (*p* < 0.0001; Figure 5D).

### 3.5 DM506 and TBG Did Not Affect Locomotor Activity of Mice

At the highest active dose used in our pain studies, acute administration of DM506 or TBG did not alter locomotor activity in mice. Mice were injected with either DM506 (40 mg/kg, i.p.) or TBG (40 mg/kg, i.p.), and locomotor activity (for 60 min) was measured at 6 and 24 h (for DM506) and 3 and 24 h (for TBG). The two-way ANOVA analysis showed no significant effect of treatment with DM506 [F_(1, 14)_ = 1.2859; *p* = 0.2808, Figure 6A] or TBG [F_(1, 14)_ = 0.388; *p* = 0.5438, Figure 6B].

**Figure 6:**
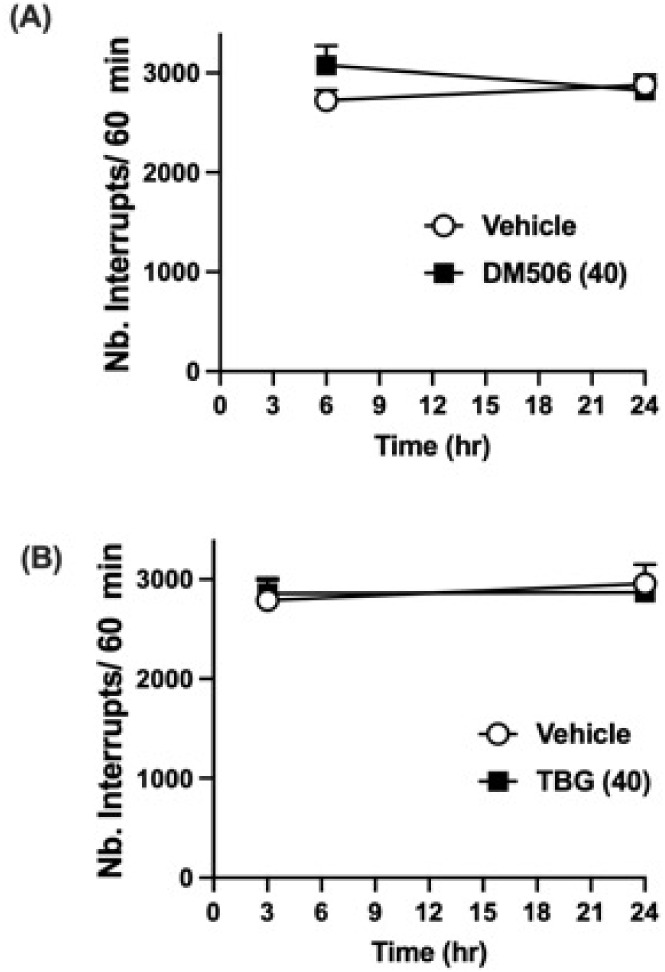
Effects of DM506 and TBG on the locomotor activity of mice. Intraperitoneal administration of (A) DM506 fumarate (40 mg/kg) or (B) TBG fumarate (40 mg/kg) did not alter the locomotor activity of mice at 3 h (TBG), 6 h (DM506), and 24 h (both) after treatment. Values are expressed as mean ± SEM, *n* = 8/group (F+M).

All together, these results show that DM506 and TBG reverse paclitaxel-induced nociceptive behaviors in a time- and dose-dependent manner via a mechanism involving 5HT_2A_R activation with no change in locomotor activity or hallucinogenic-like effects in mice.

### 3.6 Effects of DM506 on Paclitaxel Neurotoxicity

As shown in Figure 7A, DM506 showed partial neuroprotection *in vitro* against paclitaxel neurotoxicity in primary rat DRG neurons. A one-way ANOVA analysis of the results demonstrated a significant effect [F_(9, 40)_ = 7.214; *p* < 0.0001]. At concentrations of 100 nM and 300 nM, it prevented reduction in nicotinamide adenine dinucleotide induced by paclitaxel, an observation that has been shown to play a key role in programmed axon degeneration.^22^ However, at 1 μM and higher concentrations, this neuroprotection was lost. The partial neuroprotective effect of DM506 was blocked by volinanserin pretreatment (including F and *p*-values [F_(9, 40)_ = 21.11; *p* < 0.0001]) (Figure 7B), supporting a role for the 5-HT_2A_R.

**Figure 7:**
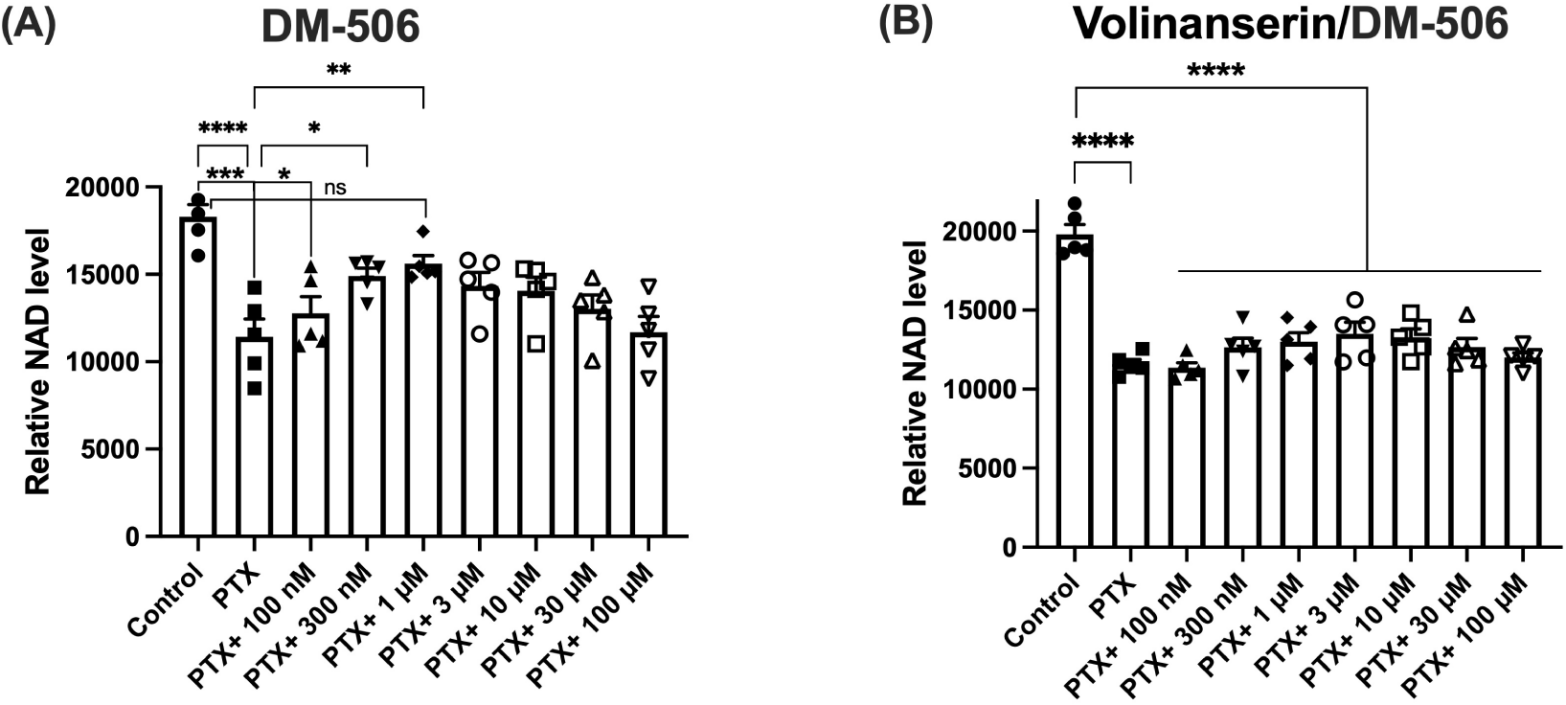
Effects of DM506 on Paclitaxel-Induced Peripheral Neuropathy. Rat DRG neuron cultures were exposed to PTX (100 nM) with or without DM506 (A) at multiple concentrations. (B) Effects of volinanserin on DM506’s activity. Cellular NAD levels were measured using mass spectroscopy and normalized to cell numbers (*n* = 5). Statistical analysis was done by one-way ANOVA with correction for multiple comparisons. Stars denote statistical significance compared to control or paclitaxel (PTX), * *p* < 0.05, ** *p* < 0.01, *** *p* < 0.001, **** *p* < 0.0001. NAD = nicotinamide adenine dinucleotide. Values are expressed as mean ± SEM.

### 3.7 Effects of DM506 on Proinflammatory Molecules in C20 Microglial Cells Treated with Paclitaxel

We assessed whether DM506 modulates the expression of cytokines IL-6 (Figure 8A) and CCL2 (Figure 8B) in C20 microglial cells in the absence and presence of paclitaxel. Two-way ANOVA analysis of the IL-6 results showed that there was not a significant main effect of stimulus (i.e., paclitaxel) [F_(1, 37)_ = 1.260; *p* = 0.27] or drug (i.e., DM506) [F_(3, 37)_ = 1.598; *p* = 0.21], but there was a significant interaction between stimulus and drug [F_(3, 37)_ = 6.561; *p* < 0.002]. Fisher’s post hoc analysis revealed that IL-6 expression was significantly increased by paclitaxel (*p* < 0.01), but neither DM506 nor ketanserin significantly affected paclitaxel-stimulated IL-6 expression. In unstimulated control cells, DM506 (*p* < 0.001) and ketanserin (*p* < 0.01), either alone or in combination (*p* < 0.01), significantly induced IL-6 expression.

**Figure 8:**
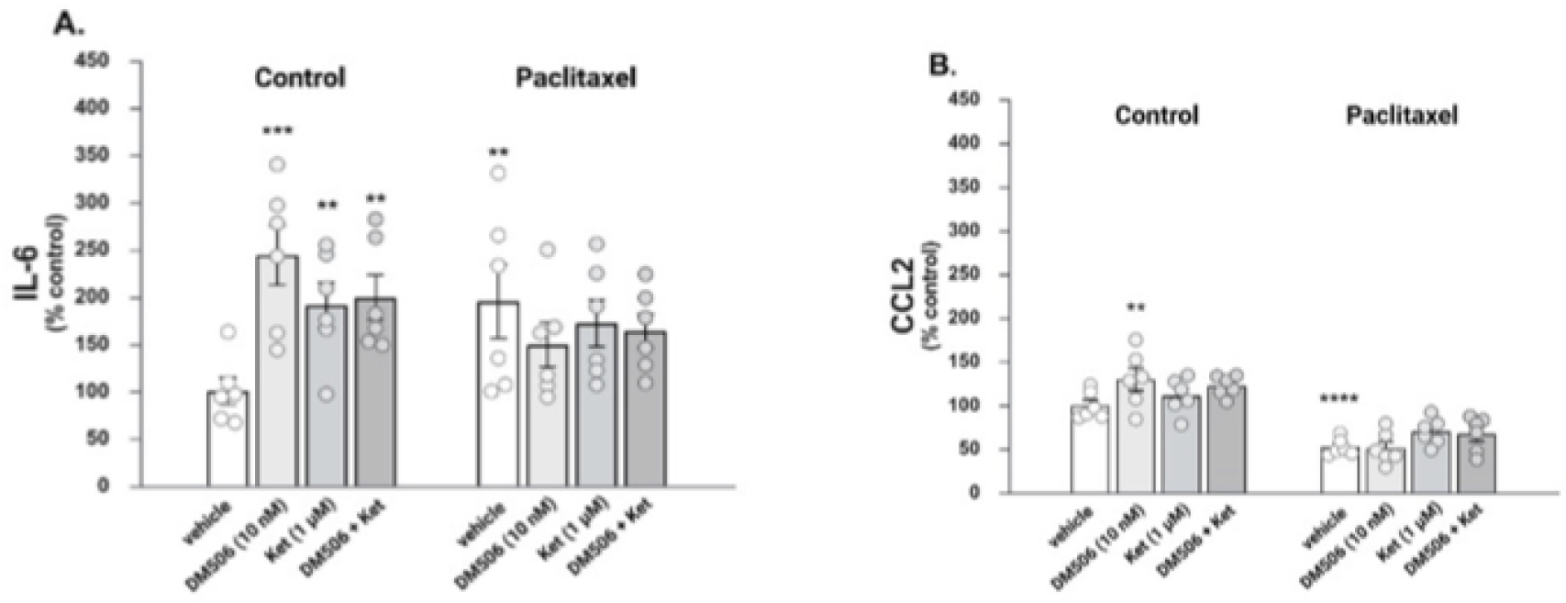
Effects of DM506 and paclitaxel on human C20 microglial cells. Unstimulated and 10 μM paclitaxel-stimulated cells (*n* = 5–6) were treated with 10 nM DM506 or vehicle for 24 h. To assess the involvement of the 5-HT_2A_R, cells were preincubated with 1 μM ketanserin (Ket) for 30 min before cotreatment with paclitaxel and/or DM506. Following drug treatment, the levels of IL-6 (A) and CCL2 (B) were measured in the culture medium using ELISA. Statistical analysis of the results (mean ± SEM) indicated that both DM506 (***p < 0.005) and Ket (**p < 0.01) significantly increase IL-6, whereas only DM506 significantly increases CCL2 (**p < 0.01) in unstimulated cells. On the other hand, paclitaxel significantly increased IL-6 (**p < 0.01) and significantly decreased CCL2 (****p < 0.001) in a DM506- or Ket-insensitive manner (*p* > 0.05).

Statistical analysis of the CCL2 expression revealed a significant main effect of stimulus [F_(1, 40)_ = 103.0; *p* < 0.0001] but no main effect of drug [F_(3, 40)_ = 2.171; *p* = 0.11] and no significant interaction between stimulus and drug [F_(3, 40)_ = 2.274; *p* = 0.095]. Fisher’s post hoc analysis indicated that CCL2 expression was significantly inhibited by paclitaxel (*p* < 0.0001), but neither DM506 nor ketanserin significantly affected paclitaxel-stimulated CCL2 expression (*p* > 0.05). In unstimulated control cells, DM506 significantly induced CCL2 expression (*p* < 0.01), whereas ketanserin, either alone or in combination with DM506, did not significantly impact CCL2 levels (*p* > 0.05). These results suggest that in unstimulated C20 microglia, DM506 increases IL-6 and CCL2 content through a mechanism different from 5-HT_2_R activation. In stimulated cells, though, DM506 does not modify the effects elicited by paclitaxel (i.e., increased IL-6 and decreased CCL2).

## 4 Discussion

The primary goal of this study was to determine in mice the activity of DM506 and TBG, two synthetic analogs of iboga alkaloids, in the CIPN mouse model, as well as to assess the role of the 5HT_2A_R and microglial cells in that effect.

Our mouse CIPN study showed that DM506 and TBG reversed paclitaxel-induced mechanical hypersensitivity in a dose- and time-dependent manner, with no observable effects on motor function or hallucinogenic-like activity in animals. More specifically, our results demonstrate that a single administration of DM506 can reverse mechanical hyperalgesia in the CIPN model between 10 and 14 days. However, the effects of TBG lasted only up to 24 h in the same model. A similar profile was also observed in the cold hypersensitivity measure. No significant sex differences in the antinociceptive effects of DM506 and TBG in the pain model were observed. Finally, our results also show that volinanserin, a selective 5-HT_2A_R antagonist, blocked the effects of DM506 and TBG, suggesting that despite differences in the time course of efficacy, the antinociceptive effects of both analogs seem to be mediated via 5HT_2A_R-dependent signaling.

Our radioligand competition binding experiments with [^3^H]­ketanserin showed that DM506 binds to the 5-HT_2A_R with high affinity (129 ± 34 nM). This affinity is relatively lower when compared to previous studies, one competing [^3^H]­LSD (*K*_i_ = 24 ± 21 nM),^7^ and another, like ours, competing [^3^H]­ketanserin (*K*_i_ = 19 ± 1 nM).^8^ Previous studies showed that DM506 binds to a high-affinity site (*K*_i_ = 19 ± 1 nM), activating the 5-HT_2A_R with high potency (EC_50_ = 9.0 ± 1.8 nM) but partial efficacy (E_MAX_ = 76 ± 16%).^6–8^ In comparison, the values for TBG were *K*_i_ = 7.2 μM,^24^ EC_50_ = 127 ± 22 nM, and E_MAX_ = 91 ± 18%.^8^ As for potential hallucinogenic activity, our experiments examining head twitch response were similar to previous studies with DM506 and TBG,^3,5,6^ eliciting no effects, indicating that these ibogalogs do not induce hallucinogenic activity at the active doses tested.

The effects of DM506 on nociceptive evoked behaviors in the CIPN model were dose-dependent and lasted 14 days. However, the effects of TBG lasted only 24 h after the injection. Interestingly, we found the effects of both DM506 and TBG in our CIPN chronic pain model are mediated by the 5-HT_2A_R subtype. Despite the assumed similarity between these two compounds (i.e., 5-HT_2A_R mediating their effects in our pain model), our results suggest that caution should be used when attempting to generalize results between different 5-HT_2A_R nonpsychedelic agonists given their complex pharmacological profiles. Indeed, in a recent study of various cryo-EM structures of different 5-HT_2A_R, it was shown that psychedelic and nonpsychedelic agonists stabilize varying conformational ensembles of the receptor^23^ which could lead to different molecular signaling.

The antinociceptive activity of DM506 and TBG in the paclitaxel CIPN model showed similar efficacy to that observed for the neuropathic pain (chronic constriction injury or CCI and oxaliplatin models) and visceral pain models (dextran sulfate sodium model or DSS) in mice.^7,8^ While the time-course effects of TBG in that study were similar to our results, DM506’s one was different. DM506 reversal of mechanical and cold hypersensitivity in the paclitaxel CIPN model lasted up to 14 days after single administration in our studies, while it only lasted 90 and 240 min in the CCI and DSS models, respectively.^7,8^ While this difference in time course can be attributed to the use of different neuropathic pain models (CIPN vs CCI), different chemotherapy agents (paclitaxel vs oxaliplatin), or mouse strains (C57BL/6J vs CD-1 and C57BL/6N strains), we believe that the doses of DM506 used in these studies played an important role (10 and 40 mg/kg in our study vs 5 and 15 mg/kg in 5 and 8). Indeed, as recently reported, DM506’s effects after a single administration of 25 mg/kg lasted more than 3 days in depression-like mouse models.^6^ Another possibility is that DM506 lasts a longer time than TBG in the brain (∼3 h) before being metabolized.^3^ However, it has been recently demonstrated that TBG can produce sustained antidepressant-like activity through a mechanism involving 5-HT_2A_R-induced neuroplasticity.^24^ Another potential explanation for the longer activity of DM506 compared to that for TBG is that the former ibogalog activates, whereas the latter inhibits, the 5-HT_2B_R.^8^ This hypothesis is supported by previous studies showing that 5-HT_2B_R activation reduces allodynia for 17 days, whereas the selective antagonist RS127445 prevents this effect.^25^

The antinociceptive effects of DM506 and TBG occurred without any impact on the locomotor activity of the mice. We assessed locomotor activity in mice after injection of DM506 and TBG at the highest tested dose (40 mg/kg) and found no significant effects on locomotion compared to vehicle-treated mice at times between 3 and 24 h postinjection. In a recent study with DM506 at the same dose of 40 mg/kg, a reduction of locomotor activity was observed in mice but only during the first 30 min.^5^

To determine whether ibogalogs modulate proinflammatory molecules in microglial cells, the effect of DM506 on the expression of cytokines such as IL-6 and CCL2 was determined in human microglial C20 cells treated with paclitaxel. The results showed that paclitaxel increases IL-6 and decreases CCL2 in a DM506/ketanserin-insensitive fashion, whereas DM506 increases both cytokines in a paclitaxel/ketanserin-insensitive manner. The first conclusion is that paclitaxel and DM506 stimulate microglial cells by different mechanisms and that DM506 does not counteract paclitaxel’s effects. Another possible conclusion is that the observed antineuropathic activity of DM506 is mediated by an anti-inflammatory mechanism that does not necessarily involve microglial cells. The cytokine IL-6 is physiologically important to CNS function^26^ but has also been implicated in the neuropathogenesis of inflammation-associated conditions, including pain, traumatic brain injury, mood and anxiety disorders, and neurodegenerative diseases.^27–30^ Our finding that DM506 increased microglial IL-6 expression, in both the absence and presence of paclitaxel, is interesting considering the evidence that this cytokine is neuroprotective in several models of neurological insult. For instance, IL-6 administration protected against paclitaxel-induced neuropathy in mice without diminishing antitumor activity^31^ and prevented paclitaxel-induced cytotoxicity in rat pheochromocytoma PC12 cells, posing the potential therapeutic effect of IL-6 in reducing chemotherapy-induced neuronal damage.^32^ Other studies found that IL-6 protected neuroblastoma SH-SY5Y cells from oxidative damage^33^ and repopulated microglia-mediated brain repair in a preclinical model of traumatic brain injury.^34^ On the other hand, 5-HT_2A_R inhibition reduced lipopolysaccharide (LPS)-induced expression of IL-6 and NF-κB, a nuclear factor that regulates cytokine’s synthesis, in macrophage cells from different origins.^35,36^ Since DM506 activates the 5-HT_2A_R,^3,5–8^ we tested the effect of ketanserin on DM506-induced activity. The results showed that DM506’s effect was not modified by ketanserin, suggesting a mechanism distinct to 5-HT_2A_R activation or even 5-HT_2B/2C_R modulation. The observed DM506-induced IL-6 expression might be part of a novel glial mechanism that reduces neuropathic pain without the involvement of 5-HT_2A_R activation. An alternative mechanism might involve the induction of the brain-derived neurotrophic factor (BDNF) intracellular pathway in microglia,^37^ as observed with other psychedelics.^38^ Considering that activation of the 5-HT_7_R in microglial cells may increase IL-6 (reviewed in ref. 39), whereas DM506 behaves as an inverse agonist at this receptor,^7^ we can rule out this receptor subtype as an important participant. On the other hand, DM506 increased CCL2 in the absence and presence of paclitaxel. Different studies have highlighted the role of CCL2 in inflammatory and neurological conditions. Although the function of CCL2 is controversial, with evidence in favor or against its beneficial effects, CCL2 may improve synaptic transmission and excitability of hippocampal neurons^40^ and regenerate peripheral nerve injury.^41^

To assess if the antinociceptive effects of ibogalogs in the CIPN model were due to neuroprotection, we assessed the effects of DM506 on neurotoxicity of paclitaxel in primary DRG neuronal cultures. Although there was a partial neuroprotection at lower concentrations of DM506 (100 and 300 nM), this was no longer seen at higher concentrations (≥1 μM), probably by an inhibitory action on α7 nicotinic receptors.^11^ This partial neuroprotection was blocked by volinanserin pretreatment, suggesting that the effect of DM506 was mediated through 5HT_2A_R activation. Although the *in vitro* results suggested that DM506 may have some neuroprotective effects at lower concentrations, it is unclear whether this played a role in the antinociceptive effects seen in the *in vivo* study. Future studies will have to evaluate a potential *in vivo* neuroprotective effect of DM506.

In conclusion, our results show that DM506 and TBG induce antinociceptive effects in a mouse CIPN model via 5-HT_2A_R activation without necessarily involving microglial anti-inflammatory mechanisms. These results, along with recent studies,^7,8^ support the development of ibogalogs as potential therapeutic targets for chronic pain management.

## Data Availability

The data that support the findings of this study are available from the authors upon request.
